# Psychometric properties of the Spanish version of the once-daily Urticaria Activity Score (UAS) in patients with chronic spontaneous urticaria managed in clinical practice (the EVALUAS study)

**DOI:** 10.1186/s12955-019-1087-z

**Published:** 2019-01-31

**Authors:** Ignacio Jauregui, Ana Gimenez-Arnau, Joan Bartra, Moises Labrador-Horrillo, Javier Ortiz de Frutos, Juan Francisco Silvestre, Joaquin Sastre, Manuel Velasco, Marta Ferrer, Cristina Ballesteros, Antonio Valero

**Affiliations:** 1 Allergy Department, Hospital Universitario Basurto, Bilbao, Spain; 2 Dermatology Department, Hospital del Mar, Parc de Salut Mar, Universitat Autonoma Barcelona, Barcelona, Spain; 3 Allergy Unit, Pneumology Department, Hospital Clinic, University of Barcelona, Barcelona, Spain; 4 Institut d’Investigacions Biomediques August Pi i Sunyer (IDIBAPS), Barcelona, Spain; 5 Allergy Section, Department of Internal Medicine, Hospital Vall d’Hebron, Universitat Autonoma de Barcelona, Barcelona, Spain; 6 Dermatology Department, Hospital Universitario 12 de Octubre, Madrid, Spain; 7 Dermatology Department, Hospital General Universitario de Alicante, Alicante, Spain; 8 Allergy Department, Fundacion Jimenez Diaz, Madrid, Spain; 9 Dermatology Department, Hospital Arnau de Vilanova, Valencia, Spain; 10 Department of Allergy and Clinical Immunology, Clınica Universidad de Navarra, Pamplona, Spain; 11 Novartis Farmacéutica, Barcelona, S.A Spain; 12 Servicio de Alergia, Hospital Clínic de Barcelona, Carrer de Villarroel 170, 08036 Barcelona, Spain

**Keywords:** Chronic urticaria, Questionnaire, Psychometric, Reliability, Validity, Spanish

## Abstract

**Background:**

The daily diary Urticaria Activity Score (UAS) and its weekly score (UAS7) are widely used to assess signs and symptoms in patients with chronic spontaneous urticaria (CSU). The objective of this study was to assess the psychometric properties of a Spanish version of the once-daily UAS.

**Methods:**

Observational study in patients ≥18 years old receiving usual care for CSU (daily or almost daily occurrence of generalized hives or angioedema for ≥6 weeks). Patients were included consecutively and completed the UAS, EQ-5D, and the Chronic Urticaria Quality of Life scale (CU-Q_2_oL) at two study visits 6 weeks apart. On each occasion, the UAS was completed once-daily for 7 consecutive days to be able to calculate the UAS7 score. Psychometric properties of reliability, construct validity, and responsiveness were assessed. The Minimal Important Difference (MID) was estimated for the UAS7 using anchor- and distribution-based approaches.

**Results:**

Data from 166 patients was available for analysis (mean age 49 years, 65.7% female). Floor (5.4% of patients with the lowest possible score) and ceiling (1.2%) effects were low; 15% of patients had missing values. Internal consistency and test-retest reliability were good (Cronbach’s alpha of 0.83 and an ICC of 0.84, respectively). Convergent validity was demonstrated through the pattern of correlations with the EQ-5D and CU-Q_2_oL and known groups’ validity was demonstrated by the instrument’s ability to discriminate between patients with different overall levels of urticaria severity, with between-group effect-sizes (ES) ranging from 0.36 to 1.19. The UAS7 proved responsive to change with effect sizes ranging from 0.3 to 1.52 in patients reporting improvement or deterioration in overall urticaria status. The MID for the UAS7 score was estimated at 7–8 points, on a scale of 0–42.

**Conclusions:**

The Spanish version of the UAS score has demonstrated a robust psychometric performance in patients with CSU managed in conditions of usual care. It can therefore be considered a suitable instrument to assess disease activity in clinical practice in Spanish-speaking patients. The Spanish version’s reliability and validity are similar to those reported for other language versions of the once- and twice-daily variants of the UAS.

## Background

Chronic urticaria (CU) is a mast-cell-driven disease characterized by the development of hives, angioedema, or both. The chronic form of the disease is differentiated from the more acute form by duration, with CU typically characterized by the development of repeated episodes of hives for more than six weeks [1]. Chronic urticaria can be classified as spontaneous (or idiopathic) or as inducible and several sub-types of urticaria exist and may co-present. Prevalence of CU in the Spanish population has been calculated at approximately 0.6% with a significantly higher prevalence in women than in men [2]. Approximately 9% of cases were found to last from one to 5 years and 11.3%, for more than 5 years [2].

Several studies have shown that urticaria can have a significant negative impact on patients’ health-related quality of life (HRQOL), with patients reporting problems attributable to their skin condition in many areas of everyday life, including personal care, recreation and social interaction, mobility, sleep, and work [3–5]. In comparison with reference groups from the general population, patients with CU scored worse on all domains of the SF-36 instrument as well as having poorer scores on several domains than patients with respiratory allergy. Their scores on a life satisfaction scale were also affected [6].

The Urticaria Activity Score (UAS) is a commonly used patient reported outcome (PRO) measure that assesses the symptoms (itch) and signs (hives) of CSU. The UAS7 score is calculated as the sum over 7 days of the daily intensity of itch (itch severity score) and number of hives score (range: 0–42), with higher scores denoting greater disease activity [7, 8]. The UAS7 has been recommended by the guidelines for use in clinical practice to determine disease activity and response to treatment [7]. Currently, the UAS7 score can be computed using the twice-daily or the once-daily UAS questionnaires. The once-daily UAS was validated in German patients in an observational study [9] while the twice-daily UAS patient assessment has been accepted by the United States’ Federal Drug Administration (FDA) as a PRO supporting a label claim for drugs in CSU and validated as per FDA guidelines for PRO instruments using data from several clinical trials [10, 11]. As part of the validation study of the twice daily version of the UAS, the Minimal Important Difference (MID) was calculated in patients with chronic spontaneous urticaria (CSU) treated with omalizumab or placebo in a randomized, double-blind, placebo-controlled trial [11] and confirmed in a later study [12].

To date, the UAS once-daily version and corresponding UAS7 has not been adapted and validated for use specifically in a Spanish-speaking population and no MID has been estimated for that version in conditions of usual clinical practice. It is important to test the psychometric properties of linguistically adapted versions of a questionnaire to guarantee that the new version shows adequate reliability, validity and responsiveness. The objective of this study was therefore to evaluate the psychometric properties of a Spanish version of the once-daily UAS and the corresponding UAS7 in patients with CSU managed according to usual clinical practice and to provide an estimate of a MID for that version. We hypothesised that the Spanish version of the UAS and UAS7 would show internal consistency and test-retest reliability coefficients over 0.70 wich is a good indicator of reliability, good convergent validity with other disease-specific and generic measures of HRQOL, ability to discriminate between groups defined by patient self-ratings of overall urticaria severity and clinician ratings of the disease, and that it would be responsive to self- and clinician-perceived change in disease activity.

## Methods

### Study design and patient population

This was an observational, prospective, multicentre study conducted in the dermatology and allergy departments of several Spanish hospitals under conditions of usual clinical practice. Data collection was performed between October 2013 and May 2014. Patients were included consecutively in the study if they were ≥ 18 years of age with a diagnosis of CSU, defined as the daily or almost daily occurrence of generalized hives or angioedema for at least 6 weeks prior to inclusion. Patients were excluded from the study if they had acute urticaria, urticaria vasculitis or other forms of urticaria not associated with the chronic form of the disease, any form of inducible CU that was not associated with CSU, angioedema without the presence of hives, pruritus related to dermatitis or other skin disease, any systemic disease or other conditions which might hinder data collection or interpretation. All patients included in the study gave their written informed consent to participate. Patients were followed up for a period of 6 weeks from inclusion in the study and were managed according to the criteria of the attending physician following their usual practice.

### Patient-reported outcomes measures (PROM)

The once-daily UAS measures urticaria activity in terms of itch severity and number of hives over the past 24 h. Response options for itch severity are 0 = None, 1 = Mild (present but not annoying or troublesome), 2 = Moderate (troublesome but does not interfere with normal daily activity or sleep), 3 = Intense (severe itching, which is sufficiently troublesome to interfere with normal daily activity or sleep). Response options for hives are 0 = None, 1 = Mild (< 20 hives/24 h), 2 = Moderate (20–50 hives/24 h), 3 = Intense (> 50 hives/24 h or large confluent areas of hives). Scores on these two items are summed to create a total daily UAS score (range: 0–6 points). A Spanish version of the instrument was produced for use in the present study following standard procedures of translation and cultural adaptation of patient reported outcome (PRO) measures [13]. The version is shown in Appendix.

Patients completed the UAS over 7 days following the first visit and in the week prior to the second visit. Patients were asked to complete the questionnaire just before going to bed in the evening. Summing daily non-missing values UAS scores over the seven-day period provides the UAS7 score, with a score range from 0 (no activity) to 42 (most intense activity).

Other PROs included in the study were the Chronic Urticaria Quality of Life scale (CU-Q_2_oL) and EQ-5D-3 L. The CU-Q_*2*_oL is a disease-specific HRQOL questionnaire for use in patients with chronic urticaria [14]. It consists of 23 items assessing HRQOL in 6 dimensions: itching (2 questions), swelling (2 questions), impact on daily activities (6 questions), sleep problems (5 questions), limitations (3 questions) and aesthetic problems (5 questions). Questions are answered on a 5-point Likert scale and scores are transformed to a 0 to 100 scale, with higher scores indicating poorer quality of life. The questionnaire generates both an overall score and scores by dimension and has been validated in Spanish [15].

The EQ-5D-3 L is a widely used generic questionnaire designed to assess health status in a wide range of conditions in adult populations [16]. It consists of 5 dimensions (mobility, personal care, daily activities, pain/discomfort, and anxiety/depression) with three possible levels of response in each dimension (absence of problems, moderate problems, extreme problems) to assess self-perceived health status on the day of completion. Utility indices based on the preferences of the general population for each of the 243 states defined by the descriptive system provide a summary score of self-rated health on the 5 dimensions and are available for various countries. They provide values ​​on a scale anchored at 0 (death) to 1 (full health) and the Spanish value set was used to calculate the utility score in the present study [17]. Patients also assess their overall health on a vertical visual analogue scale (EQ-VAS) from 0 to 100 where 0 represents the worst imaginable health state and 100 represents the best imaginable health state. The validated Spanish version of EQ-5D was used in the present study [18].

The Physician’s In-Clinic UAS, which provides a rating of the patient’s itching and hives (measured on a 0–6 scale), was also completed by the attending clinician, with input from the patient, at both study visits. Furthermore, a 5 item Likert scale with responses of ‘Very mild’, ‘Mild’, ‘Moderate’, ‘Severe’, and ‘Very severe’ was used to obtain the clinician and patient’s opinion on the overall severity of their CSU in both study visits. In the final visit, a categorical scale (Global Index of Change, or GIC) was completed by physicians and patients to obtain their opinion of the evolution of the CSU over the study period. The GIC consisted of 13 response options ranging from ‘Very much worse’ to ‘Very much improved’. Patient opinions on the ease of completion and relevance of the scale for assessing their urticaria were also evaluated using a 5-point Likert scale.

Additional study variables collected included age, sex, weight and height, ethnicity, educational level, presence of chronic spontaneous urticaria (CSU), presence of other types of urticaria associated with CSU, years since onset of symptoms, years from diagnosis, number of episodes in the previous year, presence of angiodema, number of exacerbations in the previous year, whether receiving treatment or not, presence of co-morbidities associated with CSU, history of other atopic diseases, exacerbation of CSU through NSAIDs, thyroid pathology, and associated autoimmune conditions.

### Sample size

Sample size was calculated to be able to detect a change equivalent to 0.5 standard deviations in the overall score of UAS7 (score range 0–42) between the two study visits with the aim of being able to test the instrument’s responsiveness. It was estimated that a minimum of 128 patients would be required for validation of the Spanish version of UAS7. A further 20% was added to take account of possible loss to follow-up and non-usable data to give a total sample of approximately 150 patients.

### Statistical analysis

Descriptive analysis of all variables was performed using absolute numbers and frequencies in the case of categorical variables and means or medians together with standard deviations or interquartile ranges in the case of continuous quantitative variables. The chi-square test was used to study the relationship between qualitative variables. Parametric (Student t test or ANOVA) or non-parametric (U-Mann Whitney or Kruskal Wallis) tests were used to study the relationship between continuous variables, depending on whether distribution was normal or non-normal. A statistical significance level of *p* < 0.05 was used in all analysis. Statistical analyses were carried out on the study population with valid data, without any type of imputation of missing data.

The feasibility and psychometric properties (reliability, validity, and responsiveness) of the UAS were analysed using a variety of approaches.

Feasibility was assessed by analysing the proportion of missing or unusable responses per item at each visit as well as the proportion of patients with at least one missing or unusable response. We also calculated floor and ceiling effects, i.e. patients scoring the minimum (0) and maximum scores for the UAS and UAS7 (6 and 42, respectively) scores at each visit. Floor and ceiling effects under 15% are usually considered acceptable [19].

The reliability of the questionnaire was assessed by estimating internal consistency (i.e., the degree of homogeneity of the 2 items forming the scale) and by assessing test-retest reliability (i.e., the degree of stability of the score when there are no changes in health status) [20]. The internal consistency of the scale was evaluated by calculating the Cronbach’s alpha for the overall instrument score. Test-retest reliability was assessed by calculating the intraclass correlation coefficient (ICC) for patients who, in the second study visit, declared that their urticaria symptoms had not changed from baseline using the global change item. For both Cronbach’s alpha and the ICC, a value of 0.7 or more was considered to indicate an acceptable level of reliability [21].

Construct validity, i.e. the degree to which the instrument performs as expected in meeting a series of pre-defined hypotheses, was assessed by evaluating its convergent and discriminant validity, as well as its known groups’ validity.

Convergent and discriminant validity refer to whether the instruments shows expected patterns of correlations with instruments measuring similar (convergent) and dissimilar (discriminant) constructs. They were assessed here by analysing the correlations of UAS7 scores with scores on the EQ-5D-3 L and CU-Q_*2*_oL at baseline. We also analysed the correlations between individual items (itch and number of hives) on the UAS and dimension scores on the CU-Q_*2*_oL. A series of hypotheses were developed regarding the expected pattern of correlations between the different instruments. The UAS7 score was expected to show lower correlations (r between 0.2 and 0.5) with the EQ-5D-3 L Index and VAS than with the CU-Q_*2*_oL global score, as the latter is a condition-specific measure. Higher correlations were expected between the UAS7 score and the EQ-5D-3 L dimensions of usual activities, pain/discomfort, and anxiety/depression than with the dimensions of mobility and self-care, as the former were considered more likely to be affected by CSU. On the CU-Q_*2*_oL, we expected moderate to high correlations (r = 0.5–0.8) between the UAS7 score and all dimensions of the CU-Q_*2*_oL, but we expected particularly high correlations between the itch items on the two questionnaires.

Known groups’ validity was assessed by determining the extent to which the instrument discriminated between patients according to their self-rating on the global CSU severity scale. The comparison was performed using ANOVA and between group effect sizes, which were calculated as the difference between group means divided by the pooled standard deviation. Effect sizes were classified as small (ES of approximately 0.20), moderate (ES of 0.50) or large (0.80 or more) [22]. In addition, scores were calculated for the UAS7 according to the clinician rating on the In-Clinic UAS at the first visit using the categorization proposed by Mathias et al. of < 4, 4, 5, 6 points [11].

Responsiveness was investigated by analysing the magnitude of the change in the UAS7 score corresponding to different levels of patient- and clinician-reported improvement on the global urticaria rating scale. The global index of change was also used for this analysis.

Finally, the MID for the UAS7 score was estimated in several ways, using both anchor- and distribution-based approaches [23]. For the anchor-based approach, clinician and patient ratings of the overall severity of CSU were used as the reference and a linear regression model was constructed to estimate the score change on the UAS and UAS7 corresponding to a one category shift on the scale between the two visits. Patient ratings on the GIC were also used as an anchor in an alternative model. Distribution-based MIDs were estimated based on the standard error of measurement (SEM) and standardised effect size (SES) using values of 1 SEM and 0.5 SES, as recommended [23].

## Results

Data from a total of 166 patients meeting inclusion criteria were available for analysis. All patients were recruited consecutively as specified in the study protocol.

Table 1 shows the demographic and clinical characteristics of the study population at baseline: 65.7% were female and mean (SD) age was 48.8 (14.3) yearsPatients had a median of > 2 years with symptoms of CSU and 96% was being treated for CSU at the time of the visit.

**Table 1:** Study population baseline demographic and clinical characteristics

	Baseline (*n* = 166)
Sex, female, n (%)	109 (65.7%)
Age (years), mean (SD)	48.8 (14.3)
Body mass index, mean (SD)	26.6 (4.6)
Education, n (%):	
No formal education	5 (3.2%)
Primary	56 (35.4%)
Secondary	59 (37.3%)
Higher	38 (24.1%)
Urticaria associated with CSU	65 (39.6%)
Symptomatic Dermographism	49 (75.4%)
Delayed pressure	19 (29.2%)
Heat	7 (10.8%)
Cholinergic urticaria	10 (15.4%)
Years since first appearance of symptoms, mean (SD)	2.2 (0.1-51.3)
Years since diagnosis of urticaria, mean (SD)	1.1 (0-24.2)
Nº of episodes in the prior year, median (range)	2 (0-36)
Angioedema during any episode, n (%)	77 (47.0%)
Receiving treatment for CSU, n (%)	153 (96.2%)
Patients with comorbidities associated with CSU, n (%)	85 (51.5%)

*SD* standard deviation, *CSU* chronic spontaneous urticari

### Score distributions, feasibility, and reliability

Table 2 shows the data relating to the instrument’s feasibility and reliability, as well as the mean scores and score distributions at baseline. Mean (SD) for the UAS7 at baseline was 16.4 (10.5) and there were 15% of patients with missing values. Ninety-one percent of patients found the UAS-7 ‘easy’ or ‘very easy’ to complete while 71% considered it ‘appropriate’ or ‘very appropriate’ to measure their health status in relation to their urticaria. Floor and ceiling effects were 5.4 and 1.2%, respectively. Cronbach’s alpha for the UAS7 score was 0.83 and the intra-class correlation coefficient in the test-retest sample was 0.84.

**Table 2:** Score distributions, feasibility, and reliability of the UAS7 score

	Hives	Itching	UAS7
Baseline scores and distributions			
Mean (SD)	7.2 (5.8)	9.3 (5.6)	16.4 (10.5)
Median (range)	7 (0-21)	8 (0-21)	15 (0-42)
Missing values; floor and ceiling effects, n (%)			
Missing	21 (12.7%)	21 (12.7%)	25 (15.1%)
Minimum score (floor)	22 (13.3%)	9 (5.4%)	9 (5.4%)
Maximum score (ceiling)	4 (2.4%)	6 (3.6%)	2 (1.2%)
Reliability			
Internal consistency assessed using Cronbach’s alpha (95% CI)^a^			0.83 (0.721 – 0.895)
Test-retest reliability, asessed using ICC (95% CI)			0.84 (0.735 – 0.906)

*SD* standard deviation, *Min* minimum, *Max* maximum, *ICC* Intraclass correlation coefficient. *95% CI* 95% confidence interval. Missing values defined as number and proportion of patients with at least one missing response. ^a^Test-retest reliability analysis performed in patients reporting no or minimal change in urticaria status at visit 2

### Convergent and discriminant validity

The pattern of correlations between the UAS and EQ-5D and CU-Q_*2*_oL at baseline was generally as expected (Table 3). Convergence with the generic instrument was somewhat weaker than with the disease-specific measure. Almost all of the correlations with the CU-Q_*2*_oL were statistically significant both between summary scores and at the level of individual dimensions, and correlation coefficients tended to be higher than those with the EQ-5D. The strongest correlations between the CU-Q_*2*_oL and the UAS were seen between the UAS itching item and the CU-Q_*2*_oL itching item (as expected) and summary score (0.574 and 0.628, respectively). On EQ-5D, as expected, the highest correlations were with the dimensions of pain / discomfort, usual activities, and anxiety / depression, but only the UAS itch item and overall score showed statistically significant correlations with the EQ-5D.

**Table 3:** Convergent validity of the UAS7 score. Pearson correlation coefficients with EQ-5D and CU-Q_*2*_oL summary scores and dimensions at baseline (statistically significant correlations at *p* < 0.05 in bold)

	UAS7		
	Hives	Itch	Overall
EQ-5D-3L			
Mobility	-0.030	0.037	0.005
Self-care	0.066	0.025	0.055
Usual activities	0.147	**0.287**	**0.243**
Pain/discomfort	0.143	**0.251**	**0.218**
Anxiety/depression	0.076	**0.238**	**0.162**
EQ VAS	-0.111	**–0.299**	**–0.222**
EQ Índex	-0.148	**–0.301**	**–0.247**
CU-Q_*2*_oL			
Itch	**0.606**	**0.574**	**0.628**
Swelling	**0.289**	**0.360**	**0.334**
Impact on life activities	**0.366**	**0.474**	**0.450**
Sleep Problems	**0.286**	**0.422**	**0.383**
Limits	**0.260**	**0.395**	**0.343**
Looks	**0.168**	**0.341**	**0.265**
Overall score	**0.379**	**0.510**	**0.471**

### Known groups’ validity

Known groups’ validity was demonstrated through the instrument’s capacity to discriminate between groups classified according to patient- and clinician-perceived overall urticaria severity. UAS7 scores increased with increasing severity of urticaria and with a statistically significant linear trend (*p* < 0.0001 for between-group differences in both cases) and moderate to large between-group effect sizes (Table 4). A similar trend was seen when patients were categorised according to the clinician rating on the UAS at the first visit as < 4, 4, 5, 6, with corresponding mean (SD) UAS7 scores of 13.3 (9.3) for < 4 vs 21.6 (9.6), 19.2 (8.8), and 22.5 (14.6) for the 4, 5, and 6 categories, respectively; *p* = 0.0047 for linear trend (data not shown).

**Table 4:** Known groups’ validity for the UAS7 score: mean scores for different patient-rated categories of overall CSU severity and corresponding between-group effect sizes

	Hives	Itch	Global	Between group effect size
Very mild (*n* = 20)	2.10 (2.90)	4.26 (4.42)	6.47 (6.69)	
Mild (*n* = 32)	4.81 (5.45)	7.48 (4.43)	12.40 (8.43)	0.76
Moderate (*n* = 58)	7.74 (4.37)	9.93 (5.0)	17.62 (8.30)	0.63
Severe/intense (*n* = 28)	9.61 (5.83)	11.52 (5.88)	21.04 (11.36)	0.36
Very severe /intense (*n* = 7)	17.71 (3.35)	16.63 (5.32)	33.86 (7.84)	1.19

*p* < 0.0001 for between group differences

### Responsiveness

The results of analysing responsiveness are shown in Table 5 and are based on changes in patient ratings of their overall urticaria severity between the two study visits. Effect sizes were generally larger the greater the magnitude of change. For example, a three-level change in the global rating scale corresponded to an effect size of 1.28 on the UAS7, which would represent a large effect size. This compares to an effect size of approximately 0.2 (a small effect size) on UAS7 for those indicating no change or a one-level change in their condition overall. There was essentially no difference in the size of the change on the UAS7 score between patients reporting identical levels of overall severity at the two visits (mean [SD] change of 1.9 [8.9] points) and those who reported an improvement equivalent to a change of one category (e.g. from ‘moderate’ to ‘mild’) on the rating scale (mean [SD] change of 1.7 [7.0] points).

**Table 5:** Responsiveness of the UAS7 score: mean score changes and effect sizes based on change in patient ratings of overall urticaria severity

Difference between visits in patient rating of overall urticaria severity	Hives	Itch	Global
– 3 category change (*n* = 4)	–7.6 (5.6)	–7.2 (6.3)	–15.0 (11.8)
	ES: 1.39	ES: 1.14	ES: 1.28
– 2 category change (*n* = 16)^a^	–3.7 (5.3)	–4.3 (6.3)	–8.3 (11.2)
	ES: 0.69	ES: 0.68	ES: 0.74
– 1 category change (*n* = 47)	–1.1 (4.2)	–0.7 (5.2)	–1.9 (8.9)
	ES: 0.26	ES: 0.13	ES: 0.21
No change (*n* = 59)	–0.7 (3.9)	–0.9 (4.0)	–1.7 (7.0)
	ES: 0.18	ES: 0.23	ES: 0.25
+ 1 category change (*n* = 13)	2.3 (2.6)	-0.5 (4.6)	2.3 (4.6)
	ES: 0.90	ES: 0.12	ES: 0.51
+ 2 category change (*n* = 2)	–1.5 (4.9)	1.5 (3.5)	–3.0 (8.5)
	ES: 0.30	ES:0.42	ES: 0.35

^a^For example, a two category change would mean moving from ‘moderate’ to ‘very mild’, or ‘Very severe’ to ‘Moderate’

### Minimal important difference

Distribution-based approaches to estimating a MID for the UAS7 score suggested a range for the MID between 4.4 (based on the SEM) and 5.3 (based on half of one SES). A more conservative estimate using the anchor-based approach indicates that the MID might be approximately 7–8 points based on the change in UAS7 score corresponding to a two category shift on the Global Impression of Severity scale, as shown in Table 5, and as derived from linear regression modelling, which indicated that a two category shift on the GIS would correspond to a change of 6.4 points on the UAS7. Using the same criteria, on the Itching and Hives sub-scales, the MID could be considered to be in the range of 3.5–4 points.

## Discussion

The UAS is widely used to assess CSU activity. To date, however, there is no published information relating to the psychometric performance of a Spanish version of the once-daily version of the instrument. This study remedies that and has shown that the Spanish version of the UAS has very good reliability, construct validity, and responsiveness. Given the international nature of many clinical trials and epidemiological studies today, it is of considerable importance to test and demonstrate the psychometric properties of other language versions of PROs such as the UAS and to compare results with those for other language versions.

The fact that the UAS is a relatively simple instrument may facilitate its adaptation and use in other languages as well as tending to produce relatively robust psychometric characteristics. In the present study, 91% of patients found the instrument ‘easy’ or ‘very easy’ to complete, attesting to the simple nature of the questionnaire. On the other hand, while 71% considered it “appropriate” or “very appropriate” to measure their health status and disease activity in relation to their urticaria, 30% rated the questionnaire as being of only ‘intermediate’ relevance or less. This suggests that patients might prefer other aspects of their condition to be included.

In terms of reliability, the Spanish version of the once-daily UAS shows good levels of internal consistency and test-retest reliability which exceeded the value of 0.70 which is typically considered an acceptable threshold [21]. Earlier testing of the once-daily version did not include evaluation of its reliability [9] and reliability testing of the twice-daily version showed similar results to those reported here for internal consistency but somewhat poorer results for test-reliability, with an ICC for the global UAS7 score of 0.66 [11].

The Spanish version of the UAS also demonstrated acceptable convergent validity, as our a priori hypotheses regarding the pattern of correlations between them and the EQ-5D and CU-Q_*2*_oL were generally satisfied. As predicted, correlations were higher between the UAS and the disease-specific CU-Q_*2*_oL than with the generic HRQOL measure EQ-5D. The UAS itch severity sub-scale was found to be more often statistically significantly correlated with EQ-5D dimensions and summary scores than the scale measuring number of hives, suggesting that itch is likely to have a greater impact on overall quality of life, as assessed by EQ-5D. The UAS itch severity item also showed the highest correlation with the EQ-5D pain/discomfort dimension, as would be expected. The moderate correlations observed here between the UAS7 and the CU-Q_*2*_oL also confirm Koti et al’s contention that disease activity only moderately correlates with QOL impairment in patients with CSU [24]. Interestingly, the Looks dimension of the CU-Q_*2*_oL showed the weakest correlation with disease activity though this may be connected to the location of the hives, which is something we did not investigate in the present study.

This study also provides evidence of the Spanish version’s responsiveness, with effect sizes for UAS7 scores increasing as patients indicated larger degrees of change in overall urticaria severity. The effect sizes observed in this study were somewhat smaller than those reported by Mathias et al. [11] for the twice-daily version of the UAS using data from a clinical trial. They reported SES ranging from 0.78 to 1.12 in the placebo arm and from 1.28 to 2.88 in the treatment arms. This compares with, for example, an effect size of 0.74 in our study for patients reporting a two-level improvement in their global rating of disease severity. Given that our study included a range of patients many of whom were already on treatment when they entered the study, it is perhaps to be expected that effect sizes would be smaller.

Our suggested MID of 7–8 points for the UAS7 score is slightly lower than the estimates of 9.5 to 10.5 provided by Mathias et al. based on data from several clinical trials [11, 12]. This may be due to differences in study design and to the anchors used to estimate MIDs. For example, Mathias et al. used changes on the P-UAS, Dermatology Quality of Life Index and EQ-5D as anchors, whereas we focused on overall changes in urticaria severity as perceived by patients. They also used data from clinical trials while our data was collected from patients treated in usual clinical practice. Our sample size was smaller and the number of patients in the responsiveness analysis showing a two-category change was low (*n* = 16). Additional work on a MID for the UAS7 in clinical practice would therefore be advisable.

Other limitations of the present study include the fact that responsiveness was not assessed using a before and after, interventional design. Nevertheless, based on patients’ global ratings of the severity of their condition and of change in their condition, we found evidence that the instrument is able to detect changes in patients’ urticaria status. Another limitation is that no external or objective measure of disease severity was available to provide a reference for testing of known groups’ validity, which meant we had to rely on patient and investigator ratings of overall disease severity using a single item categorical scale.

## Conclusion

The Spanish version of the UAS score has demonstrated a robust psychometric performance in patients with CSU managed in conditions of usual care. It can therefore be considered a suitable instrument to assess disease activity in Spanish-speaking patients in clinical practice. The Spanish version’s reliability and validity are similar to those reported for other language versions of the once-daily and twice-daily variants of the UAS.

## Appendix

### Spanish version of the Urticaria Activity Score (UAS)

**Instrucciones**

- Conteste a cada pregunta lo mejor que pueda.
- No hay respuestas correctas ni incorrectas.
- En cada pregunta, escoja la respuesta que mejor se ajuste a su experiencia.
- Al responder a las preguntas, piense en las **últimas 24 horas**. Marque solo una respuesta.

1. **¿Cuántos habones ha tenido en las últimas 24 horas?**

**Table:**

0	No he tenido ningún habón o roncha
1	He tenido menos de 20 habones o ronchas
2	He tenido entre 20 y 50 habones o ronchas
3	He tenido más de 50 habones o bien extensas zonas con habones confluentes o que se juntan entre ellos

*Habones o ronchas* son manchas rojas que pican, que se hinchan y que desaparecen rápidamente (en horas) en una zona del cuerpo para aparecer en otra zona del cuerpo en los sucesivos minutos u horas. Cuente cada habón por separado aunque tenga varios agrupados.

2. Valore entre 0 y 3 la intensidad del picor o prurito que ha tenido durante las últimas 24 horas

**Table:**

0	No he tenido picor o prurito
1	Leve (presente pero que no molesta)
2	Moderado (molesta pero no interfiere con la actividad diaria y con el sueño)
3	Intenso (picor o prurito grave que es capaz de interferir con la actividad diaria y con el sueño)
